# Using Natural Language Processing to Track Negative Emotions in the Daily Lives of Adolescents

**DOI:** 10.21203/rs.3.rs-6414400/v1

**Published:** 2025-04-17

**Authors:** Hadar Fisher, Nigel Jaffe, Kristina Pidvirny, Anna Tierney, Diego Pizzagalli, Christian Webb

**Affiliations:** Harvard Medical School and McLean Hospital; McLean Hospital; McLean Hospital; McLean Hospital; Noel Drury, M.D. Institute for Translational Depression Discoveries, University of California; Harvard Medical School & McLean Hospital

## Abstract

Tracking emotion fluctuations in adolescents’ daily lives is essential for understanding mood dynamics and identifying early markers of affective disorders. This study examines the potential of text-based approaches for emotion prediction by comparing nomothetic (group-level) and idiographic (individualized) models in predicting adolescents’ daily negative affect (NA) from text features. Additionally, we evaluate different Natural Language Processing (NLP) techniques for capturing within-person emotion fluctuations. We analyzed ecological momentary assessment (EMA) text responses from 97 adolescents (ages 14–18, 77.3% female, 22.7% male, N_EMA_=7,680). Text features were extracted using a dictionary-based approach, topic modeling, and GPT-derived emotion ratings. Random Forest and Elastic Net Regression models predicted NA from these text features, comparing nomothetic and idiographic approaches. All key findings, interactive visualizations, and model comparisons are available via a companion web app: https://emotracknlp.streamlit.app/. Idiographic models combining text features from different NLP approaches exhibited the best performance: they performed comparably to nomothetic models in R^2^ but yielded lower prediction error (Root Mean Squared Error), improving within-person precision. Importantly, there were substantial between-person differences in model performance and predictive linguistic features. When selecting the best-performing model for each participant, significant correlations between predicted and observed emotion scores were found for 90.7–94.8% of participants. Our findings suggest that while nomothetic models offer initial scalability, idiographic models may provide greater predictive precision with sufficient within-person data. A flexible, personalized approach that selects the optimal model for each individual may enhance emotion monitoring, while leveraging text data to provide contextual insights that could inform appropriate interventions.

## Introduction

Emotions play a fundamental role in human life, serving as essential cues that influence our actions and interactions with the environment^1^. Emotional states act as immediate alerts to potential benefits or dangers, driving us towards actions that align with our personal goals and away from potential threats^2^. Research shows that not only the intensity of emotions but also their temporal dynamics can contribute to the development of mood disorders^3–7^. This is especially important during adolescence, a developmental period marked by increases in the frequency of negative emotional states, which may contribute to elevated risk for depression^8–10^. Developing effective methods to assess daily emotional changes that are specifically suitable for adolescents is crucial. These measurements could identify both acute emotional distress and longer-term maladaptive patterns in emotions, serving two key purposes: enabling timely interventions during periods of high negative affect and facilitating targeted intervention to address maladaptive emotional patterns. Such methods could help predict increased risk of affective disorder onset (such as depression), which could in turn inform the timely and targeted delivery of preventative interventions, ultimately contributing to improved mental health outcomes.

Language offers a powerful avenue for measuring emotion, as people convey their emotional states both explicitly and implicitly through their choice of words and their manner of speaking or writing^11,12^. Language plays a dual role in the realm of emotions, serving not only as a primary tool for expressing and communicating emotional states but also in actively shaping how emotions are experienced^13,14^. Therefore, the current study aims to evaluate whether text can be used to track within-person fluctuations in emotional states.

With the rise of digital communication, people, especially adolescents, now express themselves extensively through text-based interactions, including social media posts, messages, and online discussions. This shift has generated an unprecedented volume of language data, offering a unique opportunity to study adolescents’ emotions on a larger scale^15,16^. Advances in natural language processing (NLP), a multidisciplinary field combining computer science, artificial intelligence, and linguistics, have enabled researchers to analyze these data efficiently and meaningfully^17,18^. While NLP has been used in behavioral science for decades, its accessibility and affordability have significantly improved, making it an increasingly popular tool^19^. One key advantage of NLP over traditional, manual text analysis is its ability to handle large datasets—such as thousands of social media posts or digitized texts—quickly and efficiently. By leveraging these advancements, NLP methods can identify patterns in language use (e.g., sentiment, tone, and self-referential quality), offering real-time insights into emotional states without requiring participants to actively report their feelings^20–22^. Importantly, text-based communication provides insights not only into the intensity of emotions but also into the context in which those emotions arise. This contextual information can reveal situational factors contributing to emotional experiences, offering a more nuanced understanding of emotional processes.

In recent years, various text analysis tools have emerged^23–29^, ranging from basic approaches like counting word frequencies to more advanced methods, such as leveraging large language models (LLMs), each with its own set of advantages and limitations. For example, closed-vocabulary programs (i.e., dictionary-based approach)^1^ such as the Linguistic Inquiry and Word Count (LIWC)^30^, use predefined dictionaries to categorize words. While highly interpretable, transparent, and efficient at summarizing concepts, these methods often neglect context, leading to potential misinterpretations^25^. LIWC may classify the word *mad* as an indicator of anger, yet in phrases like *“She’s mad talented,”* it conveys emphasis rather than negativity, illustrating how context-dependent meanings can lead to inaccurate classifications. In contrast, open-vocabulary approaches, such as Latent Dirichlet Allocation (LDA), and word embedding methods leverage data-driven techniques to examine a broader spectrum of words and topics^31–33^. These methods are better at capturing nuances, addressing ambiguous word meanings, and are less susceptible to misinterpretations. Limitations of open-vocabulary approaches include the need for more technical expertise, larger datasets, and careful consideration of parameter choices, as well as challenges in interpretability^25,29^.

More recently, LLMs like Generative Pre-trained Transformer (GPT) have shown great promise in accurately identifying various psychological constructs in text, overcoming many of the constraints of older methods (Rathje et al., 2024). These models can interpret the context of words and have achieved effective results across multiple languages with simple prompts^19^. The main limitations of this approach include a lack of transparency in how inferences were generated and difficulties in reproducing results due to their probabilistic nature^34^. For example, although LLM-based approaches could predict whether a person experiences negative emotions based on social media posts, it would not be possible to extract information from the LLM about which linguistic cues (such as the use of pronouns) influenced its predictions^19,27^. In the latter case, dictionary-based methods are preferable. Ultimately, closed- and open-vocabulary approaches, along with advancements in LLMs, provide complementary strengths that when combined may significantly enhance our ability to understand psychological states through language. By combining these methods, researchers may be able to leverage their unique advantages while mitigating their limitations, offering a robust alternative to traditional and relatively cumbersome self-report emotion measures that might have reporting biases.

Despite the increasing sophistication of these methods, significant gaps remain in the current literature. Most importantly, existing studies on text-based emotion prediction have focused on exploring between-person differences (nomothetic approach), primarily identifying which text features are associated with individuals experiencing high levels of negative emotions^35^. These approaches aggregate data across participants and aim to pinpoint common linguistic markers, such as the use of first-person pronouns or negative sentiment, that are indicative of heightened emotional distress and depression^36,37^. However, this method overlooks both between-person differences in how linguistic markers relate to emotions and within-person fluctuations in emotional states. Even when using multilevel models to predict within-person fluctuations in emotions, models assumes that the same linguistic features signal high levels of negative emotions across different individuals^37^.

Such assumptions fail to account for the fact that language is inherently idiosyncratic, with individuals expressing emotions in unique and context-dependent ways. Beck & Jackson^38^ demonstrated the importance of idiographic approaches, which focus on person-specific patterns, by showing that the psychological and situational antecedents that predicted future loneliness varied substantially across participants, with no two individuals showing the same pattern of predictive features. This highlights the need for personalized models that adapt to the distinctive ways people express emotions through text, enabling more accurate predictions tailored to each individual. To address this limitation, this study integrates diverse language-based tools and leverages machine learning to develop personalized models capable of monitoring within-person fluctuations in emotional states.

The study’s primary goal was to examine whether within-person fluctuations in negative emotions in adolescents’ daily lives can be accurately tracked through text analysis. Since individuals express emotions in a variety of linguistic ways, this research aims to develop more personalized approaches to emotion tracking. Specifically, we address two key questions:

1. Do idiographic (individual-level) models outperform nomothetic (group-level) models in predicting emotion fluctuations?
2. Does combining various NLP approaches improve emotion prediction?

We compare three types of Natural Language Processing (NLP) approaches, which, as noted above, have complementary strengths and limitations:

- Closed-vocabulary approach (dictionary-based approach: LIWC and VADER)
- Open vocabulary (LDA)
- GPT-derived ratings

By addressing these questions, the study aims to enhance the accuracy of emotion prediction models, potentially enabling closer monitoring of emotional fluctuations in daily life. The ultimate goal is to use these improved emotion predictions to inform the delivery of scalable, real-time, and personalized interventions for alleviating high-NA states and enhancing emotion regulation abilities in youth.

## Results

The full results can be explored in the companion web app (https://emotracknlp.streamlit.app/), which also includes an interactive chatbot powered by a GPT-based LLM. The chatbot was trained on the full manuscript and is designed to answer questions about the research, figures, and results presented in this paper, as well as additional findings.

### Demographic and clinical characteristics

Table 1 presents the demographic and clinical characteristics of the participants. For the participants who completed EMA in four consecutive weeks (see Methods for EMA schedule), the median number of EMA surveys completed was 77 (*M* = 75, *SD* = 24), and mean EMA compliance was 67% (*SD* = 21%). For the participants who completed EMA in biweekly 5-day blocks, the median number of EMA surveys completed was 66 (*M* = 68, *SD* = 24), and mean EMA compliance was 51% (*SD* = 17%).

### Model performance: Nomothetic Approach

Table 2 presents model performance metrics for predicting negative emotions using combined text features from three NLP techniques. Three modeling approaches are compared: group-level nomothetic (one model combining all observations), per-participant nomothetic (same modeling approach as group-level nomothetic but performance is calculated separately for each individual), and idiographic (separate models for each individual). Further details and results, including separate models for each NLP technique, are available in the Web app (see “Model performance across sample” and “Model performance per participant”).

The group-level nomothetic model performed well, with R^2^ values ranging from 0.11 to 0.38. However, when evaluated separately for each participant – which is critical given the ultimate goal of tracking fluctuations in emotions *within* individuals – nomothetic model performance decreased substantially. Mean R^2^ values fell between 0.06 and 0.11, with considerable variability in predictive accuracy across participants. For example, when predicting mean negative affect (NA), individual R^2^ values ranged from 0.00 to 0.41, demonstrating that while text features explained little to no variability in NA for some, they accounted for a large proportion of variability in others. See Table 2 for results for the specific negative emotions of sadness, anger, and nervousness.

### Model performance: Idiographic Approach

**Figure 1:**
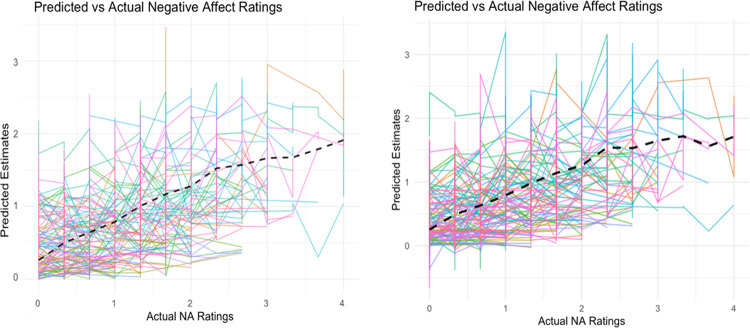
The relationship between predicted and observed (actual) negative affect ratings using person-specific (idiographic) models. Panel (a) Random Forest (b) Elastic Net. Each colored line represents an individual participant’s predicted estimates (y axis) across different levels of actual negative affect (x axis).

The idiographic approach (separate models for each individual) showed similar average performance to the nomothetic approach, with mean R^2^ values ranging from 0.06 to 0.10. However, the idiographic approach demonstrated better performance in terms of root mean squared error (RMSE) (0.47 to 0.76) compared to the nomothetic model (0.52 to 0.88), suggesting lower prediction error. Fig. 1 displays the relationship between predicted and observed (actual) NA ratings using person-specific (idiographic) models. Each colored line represents an individual participant’s predicted estimates (y-axis) across different levels of actual NA (x-axis). While the overall trend, represented by the dashed black line, suggests a generally positive relationship between predicted and actual ratings, indicating that the models capture meaningful within-person fluctuations in NA, there were large between-person differences in the strength of these associations.

**Figure 2:**
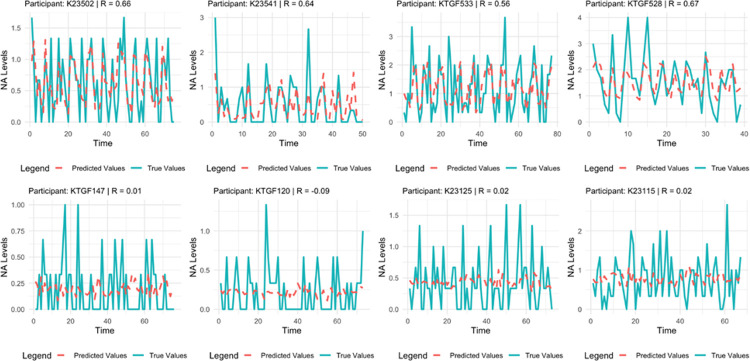
Examples of person-specific (idiographic) predictions of negative affect for high-performance (top panel) and low-performance (lower panel) models

Fig. 2 displays the substantial differences in model accuracy across individuals by showcasing examples of four high- vs low-accuracy person-specific models (figures for all other outcomes and for all participants for both the nomothetic and idiographic models are available on the Web app section “True vs. Predicted”).

### Examining Between-Person Differences in Which Text Features Predict Negative Emotions

**Figure 3:**
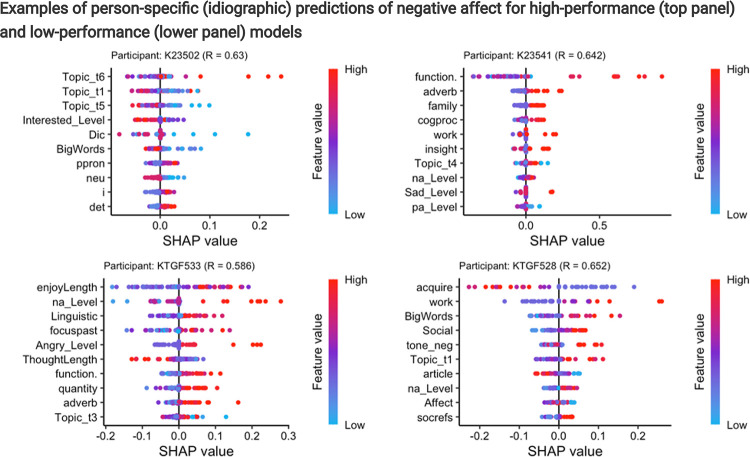
SHAP (Shapley Additive Explanations) beeswarm plots for four individual participants, illustrating the contribution of different text-based features to the model’s emotion predictions. Each dot represents an individual data point, with red indicating high feature values and blue indicating low feature values. The spread of dots across the x-axis reflects the variability in a feature’s effect on predictions across different measurement time point. The y-axis lists the features in descending order of importance, meaning the top features had the strongest impact on the model’s predictions. The x-axis represents SHAP values, indicating the magnitude and direction of each feature’s impact on the predicted emotion scores. For example, for participant K23528, a *low* score on “Acquire” (red color) increases the model’s prediction of negative affect. Conversely, a *high*feature value for “Work” or “Big Words” (red color) contributes to a higher predicted negative affect.

Fig. 3 illustrates the variability in feature importance by showing the top 10 text features from the four best-performing subject-specific random forest models predicting NA. These models are the same four high-accuracy models displayed in Fig. 2. The results show two types of variability between individuals: differences in model performance and, even among highly accurate models, differences in the text features that contribute to predicting NA. Critically, this visualization demonstrates how text features provide valuable contextual information. For example, one participant (K23541) exhibits more negative emotions when using words related to their family (“Family”), yet another (KTGF533) shows greater negative emotions when focusing on the past (“FocusPast”). Similarly, a different participant (KTGF528) expresses more negative emotions when writing about work-related topics (“Work”) but demonstrates less negativity when using words associated with acquiring objects, states, or goals that fulfill personal needs (“Acquire”). This rich information can be used to create person-specific profiles that enable us to identify the specific contexts in which negative emotions arise, providing insights that can be used to personalize interventions. A heatmap of the feature importance across participants and feature importance figures for each participant, outcome, and ML model are available on the Web app (“Feature importance heatmap” and “Feature importance per participant”). Details on the differences in predictive text features between high- and low-performing models can be found in the Supplement (**Section S4** and **Fig. S3**).

### Which NLP Approach Performs Best in Predicting Negative Emotions?

**Figure 4:**
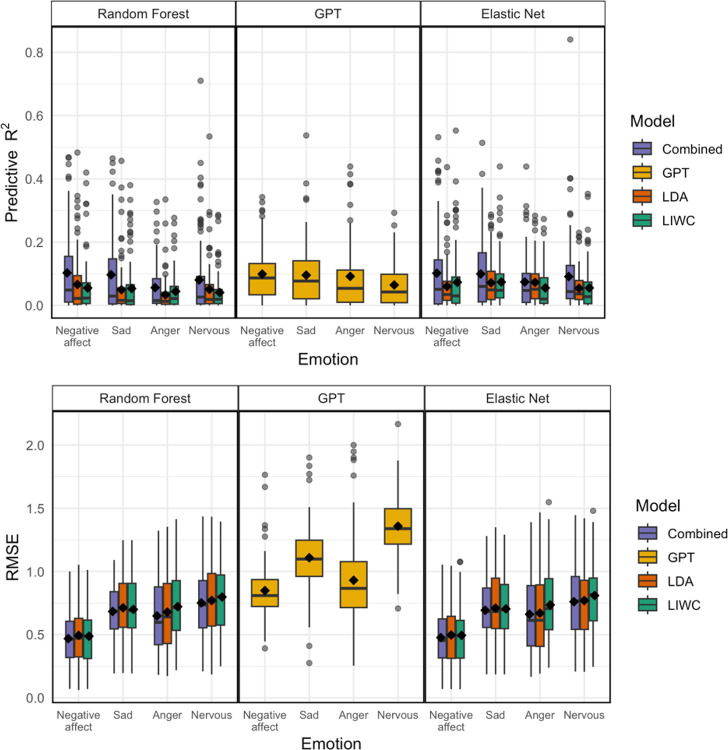
The top panel displays the predictive R^2^ for different models (Random Forest, GPT, and Elastic Net) across four emotion categories: negative affect, sadness, anger, and nervousness. The bottom panel presents the corresponding RMSE values. Each box plot represents the distribution of performance metrics for four different NLP-based feature sets: Combined (purple), GPT (yellow), LDA (orange), and LIWC+VADER (green). Higher R^2^ indicates better predictive accuracy, while lower RMSEreflects better model fit.

Table 3 and Fig. 4 displays the performance metrics for each NLP approach used in the idiographic models. When examining the idiographic predictive performance of each NLP approach separately, GPT generally showed better performance for NA, sadness, anger, and nervousness, with R^2^ values around 0.10 for NA and sadness, and slightly lower for anger and nervousness. These results were comparable to those achieved by the idiographic models that combined all NLP approaches (see Table 2 for the combined model results), except in the prediction of nervousness where GPT slightly outperformed the combined idiographic model. However, it is important to note that while GPT had relatively strong R^2^ values, it also had a higher RMSE compared to the combined models, suggesting a higher average prediction error despite accounting for more variance in the outcomes. LIWC+VADER and LDA each demonstrated poorer predictive performance, with the lowest average R^2^ values and fewer significant associations across all emotions, especially for anger and nervousness. This highlights that the contextual understanding provided by GPT was more effective in capturing within-person emotional fluctuations compared to the other methods when NLP approaches were tested individually. Overall, while GPT captures variability in negative emotions (as reflected by R^2^), its predictions are less precise in terms of exact values (as indicated by RMSE). Combined models, which balance capturing variability and minimizing prediction errors, offer a more robust and reliable approach to emotion prediction.

### Evaluating Best-Performing Models for Each Individual

**Figure 5:**
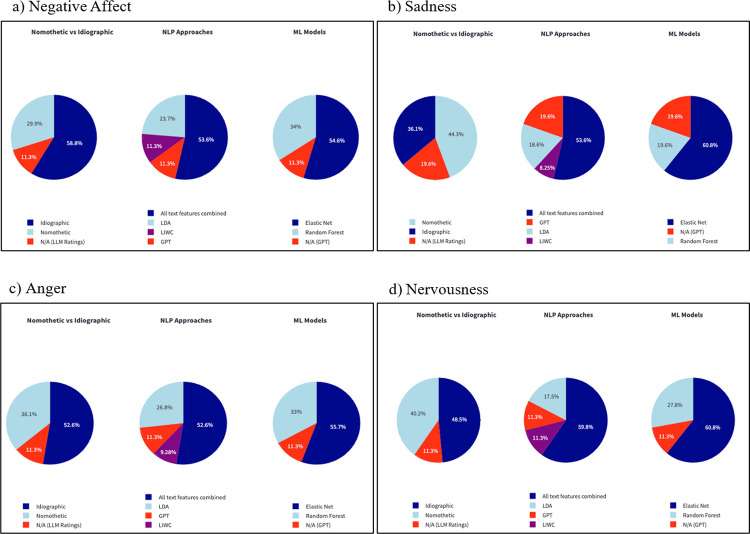
The pie charts display the distribution of best-performing models for predicting negative emotions (a: Negative affect, b: Sadness, c: Anger, d: Nervousness) across three comparison categories: nomothetic vs. idiographic models, NLP approaches, and ML models. The colors represent different model types, indicating the proportion of participants for whom each model was the best predictor.

To investigate the effectiveness of personalized models, we identified the best-performing predictive model for each of the 97 participants. Using this flexible approach, the vast majority (90.7–94.8%) of the participants showed a significant correlation between predicted and observed (actual) emotion scores. The table detailing the best model for each participant and each emotion outcome can be found in the Web app under “Best model performance.” Fig. 5 shows the distribution of best-performing models across participants. Idiographic models were most frequently selected for all outcomes except for Anger, where nomothetic models performed best for 44.3% of the participants. Elastic Net showed the best performance for most participants across outcomes, followed by Random Forest. For NLP approaches, the model combining all text features outperformed for most of the participants (52.6–59.8%). Interestingly, though GPT showed the highest mean R^2^ scores, it was chosen as the best model for only a relatively small portion of the participants (11.3–19.6%).

### Are Prediction Models Emotion-Specific?

To examine whether our models captured emotion-specific signals rather than general NA, we compared the extent to which each emotion-specific model (both Random Forest and Elastic Net) predicted each observed emotion. Specifically, for each observed emotion outcome (NA, Sad, Angry, and Nervous), we calculated R^2^ and RMSE between the observed and the predicted emotion, either matching (e.g., predicted and observed sadness) or mismatching (e.g., predicted anger and observed sadness). **Fig. S4–S7** (see also in the web app under “Best Model Performance”) display box plots of the distribution of R^2^ and RMSE for each emotion. As shown in the figures, overall, and consistent with the interpretation that the models have emotion-specific signals, the matched predictions showed higher R^2^ and lower RMSE than mismatched predictions, indicating that the models capture unique, emotion-specific variance. However, for RMSE, we sometimes observed better performance for NA relative to the specific emotions, suggesting that the signals underlying broad NA might be more robust or reliable relative to single emotion ratings.

## Discussion

In this study, we combined multiple Natural Language Processing (NLP) approaches to examine whether within-person fluctuations in emotion can be accurately tracked through text analysis. Recognizing the idiosyncratic nature of emotional communication, we compared idiographic models, tailored to individual patterns, with nomothetic models that capture common trends across groups. The leveraging of advanced NLP and machine learning techniques to track moment-to-moment emotional changes through text analysis has the potential to enhance mental health monitoring, support clinical decision-making, and enable early detection of distress for timely, personalized interventions.

The results showed that, overall, group-level nomothetic models showed high performance in continuously predicting negative emotions (R^2^ range: 0.11–0.38). These findings align with previous studies demonstrating the utility of NLP approaches in detecting emotional states across participants^39,40^. However, since these studies primarily focused on identifying emotional states at the between-person level, rather than capturing *within-person* fluctuations, their high performance does not necessarily indicate an ability to accurately track moment-to-moment emotion fluctuation within individuals. When we calculated performance metrics for each participant individually, the model performance declined significantly and showed high between-person variability, indicating that the nomothetic models’ ability to track within-person changes varies considerably from one individual to another.

When we built separate models for each participant (idiographic models) and compared their performance to that of nomothetic, we found that the mean R^2^ was comparable between the two approaches; however, RMSE was lower for the idiographic models. In addition, idiographic models revealed significant between-person variability in the text features predicting emotional states, indicating that individuals express negative emotions in distinct linguistic ways. This raises an intriguing question: if individuals differ so markedly in their predictive features, how can a nomothetic model that captures only general trends perform just as well, at least in terms of R^2^? One plausible explanation is a trade-off between statistical power and individual variation. While idiographic models capture unique, person-specific patterns, nomothetic models benefit from larger datasets that enable the estimation of multiple weaker, yet common, relationships between text features and emotions. In other words, although individual differences exist, the robust and common patterns identified by the nomothetic approach appear sufficient to achieve similar predictive accuracy. Supporting this interpretation, idiographic models showed lower RMSE and higher accuracy for more participants compared to the nomothetic models. However, nomothetic models demonstrated greater consistency across participants, with more participants presenting significant associations between predicted and actual negative emotions compared to idiographic models.

Rather than directly comparing nomothetic and idiographic models, an alternative approach is to select the best-performing model for each participant. Using this flexible approach, the vast majority (90.7–94.8%) of participants showed a significant correlation between predicted and observed emotion scores, highlighting the potential of personalized modeling strategies to improve prediction accuracy.

While we are not aware of studies directly comparing nomothetic and idiographic approaches in text analysis, previous research comparing their effectiveness in tracking mental states using passive sensor data from smartphones and actigraphy, as well as self-reported ecological momentary assessment (EMA), has yielded similar results^41–44^. For example, Aalbers *et al*.^41^ used smartphone passive sensor data to predict stress levels. Their findings revealed that idiographic models demonstrated higher accuracy in tracking stress levels for some participants (Spearman’s ρ rank-order correlation up to 1 in idiographic models vs. up to .65 in nomothetic models). However, nomothetic models significantly predicted stress for a larger proportion of participants (up to 23.2% for idiographic models vs. up to 55% for nomothetic models). Rozet et al.^43^ used a comparable method and initially found that nomothetic models performed better (i.e., were more accurate) than idiographic models. However, as more data accumulated, the performance of the idiographic model eventually equaled and then surpassed that of the nomothetic model, suggesting that idiographic models may be a better option when sufficient data is available at the single subject level.

When comparing specific NLP approaches, GPT outperformed other models in its ability to track fluctuations in emotional states. It achieved R^2^ values comparable to the model combining all three NLP approaches. Despite the relatively high R^2^ values, GPT predictions also showed higher RMSE scores compared to the combined models, suggesting that while GPT may account for meaningful variance in emotional states, it may be less precise in predicting the exact numerical values of these states. Furthermore, when selecting the best-performing model for each participant, GPT was chosen for only a relatively small portion of individuals, indicating that its predictions are **relatively stable across individuals but not necessarily the most accurate for any single participant. This pattern aligns with the nomothetic-idiographic trade-off discussed earlier, as GPT operates as a nomothetic model that generalizes across individuals rather than incorporating person-specific information**. Future research should aim to optimize this balance by leveraging fine-tuning techniques, such as **participant-specific calibration or supervised fine-tuning on emotion-labeled data**, to improve GPT model accuracy.

From an applied perspective, GPT is already trained on a vast corpus of data (approximately 45 terabytes of text data from various sources^45^), which makes it particularly attractive for studies with limited datasets, as it does not necessarily require retraining on task-specific data to yield meaningful predictions. However, the model’s lack of transparency and inability to explicitly articulate the rationale behind its predictions remain critical limitations that researchers must carefully weigh^19^. Given these considerations, we recommend that researchers thoughtfully evaluate GPT’s role in their analytical pipeline, considering whether to employ it as a standalone tool, integrate it with complementary approaches, or stick with alternatives. Future work should focus on developing methods that combine GPT’s powerful inference capabilities with more transparent analytical approaches, potentially offering a more robust framework for emotion analysis in psychological research.

### Limitation and future directions

This study is the first to use different NLP approaches to directly compare nomothetic and idiographic models for tracking emotion fluctuations. Still, our findings should be interpreted in light of several limitations. First, capturing the nuanced dynamics of emotional expression typically requires large amounts of data. In everyday interactions, subtle emotional changes may only become apparent with extensive exposure to an individual’s language use, much like knowing someone well enables you to discern small shifts in their mood. Therefore, future studies should strive to collect larger within-person datasets, potentially sourced from daily-life communications, to enhance the ability to track and model these nuances.

Second, the overall predictive accuracy was modest, leaving significant room for improvement. In addition to increasing the quantity of data, future research could benefit from incorporating additional modalities (e.g., vocal features, facial expression, passive sensors from smartphones or wearables such as smartwatches or smart rings, which are becoming increasingly popular) alongside text. Prior studies have shown that combining various modalities can enhance the performance of nomothetic models of emotion (see Gandhi et al.,^46^ for a review), and thus this multimodal approach may also improve idiographic models. Moreover, future work should explore whether individuals not only differ in the text features that predict their emotions but also in the types of modalities that best capture their emotional states. For example, individual differences, such as the tendency to use affective suppression, may influence how emotions are conveyed in text, with greater suppression potentially leading to lower model performance. Future studies should examine whether these participants require larger datasets to capture subtle nuances in their text, or if alternative modalities (e.g., passive sensor) better detect changes in their emotional states.

Third, the current study compared idiographic and nomothetic approaches, demonstrating that each has its merits. Future studies could explore hybrid approaches that integrate both individual-specific and group-level information, balancing personalization with statistical power to enhance predictive accuracy. Finally, the text data in our study consisted of responses to specific questions, which limits the generalizability of our findings to other types of text (e.g., text messages and social media posts). Future research should incorporate text from diverse sources such as social media, conversational exchanges, and free-form writing, to determine whether these results extend to broader contexts.

## Conclusion

In conclusion, our findings highlight the potential of combining NLP approaches to track within-person emotional fluctuations, demonstrating both the strengths and limitations of idiographic and nomothetic models. Nomothetic models effectively capture general trends, making them useful for broad applications. In contrast, idiographic models provide a more nuanced understanding by identifying person-specific features that reflect an individual’s unique emotional expression, though they require large within-person datasets to model these patterns reliably. Rather than a one-size-fits-all approach, our results suggest that selecting the best approach for each participant can enhance predictive accuracy. Expanding this work to incorporate diverse text sources and multimodal data streams (e.g., integrating social media posts, speech transcripts, facial expressions, physiological signals, or smartphone sensor data) may further advance the field.

Ultimately, improving the ability to monitor emotions in real-time not only enhances our ability to study emotional phenomena as they unfold in daily life but also can help inform the development and deployment of just-in-time (JIT) interventions that go beyond identifying moments of distress to also consider the specific emotions and contextual factors in which they arise, enabling more personalized and effective interventions for youth.

## Methods

### Participants

Participants were derived from two larger studies that recruited adolescents with elevated levels of anhedonia, as well as typically developing (non-anhedonic) adolescents. They included 97 English-speaking adolescents aged 12–18 (75 female, 22 male; M_*age*_ = 16.2, *SD* = 1.9) recruited from the greater Boston area. Participants were excluded based on a history or current diagnosis of any of the following DSM-5 psychiatric illnesses: schizophrenia spectrum or other psychotic disorder, bipolar disorder, substance or alcohol use disorder within the past 12 months or lifetime severe substance or alcohol use disorder. Participants were also excluded based on current diagnosis of anorexia nervosa or bulimia nervosa or if they had a neurodevelopmental disorder that would interfere with study tasks. Due to the neuroimaging component of this study, additional exclusion criteria included fMRI contraindications. For additional information about sample inclusion and exclusion criteria see Murray et al., (2023).

### Procedure

All procedures were approved by the Mass General Brigham IRB. Participants who were 18 years of age provided written informed consent; participants who were under 18 provided written assent, with their parents providing written consent. At the baseline session, either in-person or over Zoom, participants were administered a semi-structured clinical interview, the Kiddie Schedule for Affective Disorders and Schizophrenia (K-SADS; Kaufman et al., 1997) and completed self-report measures. Following the baseline session, participants installed the MetricWire app on their smartphones to complete ecological momentary assessments (EMAs). Of the participants, 39 completed the EMA for four consecutive weeks; for the first 5 days, surveys were delivered 2–3 times per day in the afternoon and evening, and for the remaining 25 days, surveys were delivered 4 times per day. The other 58 participants completed the EMA in 5-day (Thursday through Monday) blocks every other week, with 2–3 survey prompts per day, for a median of 17 weeks.

### Measures

#### Ecological Momentary Assessment (EMA)

Participants were asked to rate on a 5-point Likert scale, ranging from 0, “Very slightly or not at all,” to 4, “Extremely” the extent to which they were feeling several emotions immediately before they started the assessment. Participants’ negative affect (NA) included responses for “sad,” “nervous,” and “angry.” Mean NA was measured by averaging these three variables. In addition, participants responded to the following open-ended questions: 1) What were you thinking about right before you started this survey? 2) Think about the most enjoyable or happy time since you completed the last survey (or if this is your first survey, then the last 24 hours). Very briefly, what happened (1–2 sentences is fine)? 3) Think about the most stressful or negative time since you completed the last survey (or if this is your first survey, then the last 24 hours). Very briefly, what happened (1–2 sentences is fine)? Participants with fewer than 30 observations were excluded from the analysis.

#### Language Measures

We used the following strategies to extract text features and generate quantitative summaries of the language data.

Closed-vocabulary. For the closed-vocabulary (dictionary-based) approach, we extracted features using the Linguistic Inquiry and Word Count (LIWC)^28^ and the Valence Aware Dictionary and sEntiment Reasoner (VADER)^47^. LIWC is a computerized text analysis tool that categorizes words into over 90 linguistic and psychological dimensions based on a dictionary of approximately 6,400 words^48^. It calculates the percentage of words that match each predefined category, offering insights into linguistic structures (e.g., pronouns), psychological constructs (e.g., affect), and broader language patterns (e.g., analytical thinking). LIWC has been extensively validated across numerous studies and is widely used in psychological research to quantify language use patterns associated with various psychological states and traits. For this study, we used LIWC-22^23^, the latest version of the software, the analyze participants’ responses to the EMA open questions.

VADER is a simple rule-based model for general sentiment analysis optimized for social media. It provides four sentiment scores: negative, positive, neutral, and compound (an overall sentiment score from −1 to +1). VADER is particularly effective at handling sentiments expressed in short, informal text and accounts for factors like punctuation, capitalization, and modifiers (e.g., “very”) that influence the intensity of the sentiment. Whereas LIWC excels in offering detailed psychological and linguistic insights across a wide range of texts, VADER is more attuned to detecting sentiment polarity and intensity in social media. Finally, text lengths were extracted as features for each question and included in the models.

Open-vocabulary. Latent Dirichlet Allocation (LDA) is a probabilistic clustering method that identifies topics based on word co-occurrence rather than relying on predefined dictionaries^31,32^. This approach allows LDA to group semantically related words while considering context, reducing word sense ambiguity. The latent topics were extracted from the preprocessed text corpus using probabilistic modeling with the R package *topicmodels*^49^. In our study, we implemented LDA using two distinct approaches for nomothetic and idiographic analyses. For the **nomothetic model**, we extracted a uniform set of topics across the entire sample. For the **idiographic model**, we applied LDA separately for each individual, identifying topics based on their unique word usage patterns. To interpret the extracted **nomothetic topics**, Table 4 presents the 15 most frequent words per topic, offering a semantic representation of the clustering. Details on topic selection, model tuning, and evaluation metrics are provided in the Supplement **Section S1** and **Fig. S1**. Furthermore, **Fig. S2** depicts the distribution of LDA-derived topics across participants, highlighting variations in topic prevalence within the dataset.

Generative Pre-trained Transformer (GPT). To generate emotion ratings using large language models (LLMs), we used GPT, a deep learning-based AI language model developed by OpenAI. GPT is built on a transformer-based architecture, a neural network design that excels at processing sequential data by using self-attention mechanisms to capture contextual relationships across words. This allows GPT to generate human-like text by predicting the next word in a sequence based on the provided context. Specifically, we used GPT-4, an advanced version pre-trained on a vast dataset (45TB), enabling it to generate coherent sentences and perform various tasks such as writing, answering questions, and engaging in conversations. In this study, we prompted GPT-4 through the application programming interface (API) using Python code to rate the extent to which a participant experienced one of the following emotions: Sadness, Anger, and Nervousness, on a scale of 1 to 5 (similar to the scale used in EMA), based on responses to three open-text questions. See **Section S2** in the supplement for the full prompt used to generate GPT responses.

### Data Analysis

#### Model Specification

**Figure 6:**
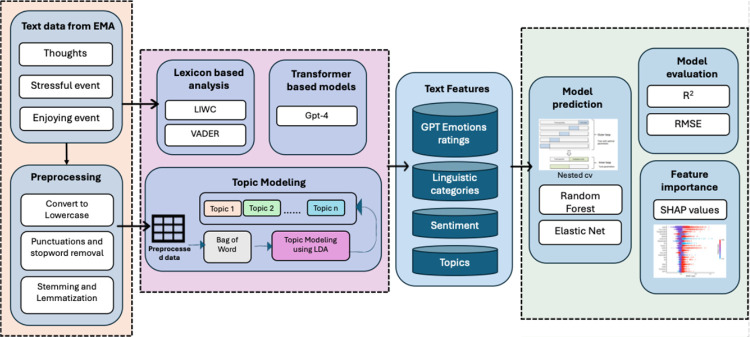
The pipeline for processing and analyzing text data from EMA responses to predict emotional states. The process begins with preprocessing (e.g., text normalization, stopword removal, stemming). Text features are then extracted using lexicon-based analysis (LIWC, VADER), transformer models (GPT-4), and topic modeling (LDA). These features, including GPT emotion ratings, linguistic categories, sentiment scores, and topics, are fed into machine learning models (Random Forest, Elastic Net) for prediction. Model performance is evaluated using R^2^ and RMSE, while SHAP values provide insight into feature importance. This approach integrates multiple NLP techniques to enhance emotion prediction accuracy.

We began our analysis by extracting text data from EMA responses and conducting both lexicon-based and transformer-based analyses. Next, we applied preprocessing steps (e.g., text normalization, stopword removal, stemming) to refine the text before performing topic modeling. Finally, we trained machine learning models to predict negative emotions based on the derived text features, evaluated model performance, and determined feature importance. Fig. 6 illustrates this analysis pipeline in detail. The code is available at: https://osf.io/grjy9/files/osfstorage.

We compared both nomothetic (group-level) and idiographic (individual-level) models in their ability to predict variability in negative emotional states *within* individuals over time. We employed two machine learning approaches to predict negative emotions: . Elastic net regularization (ENR) and Random Forest models (RF). ENR is a popular variant of conventional regression that combines two types of penalties: ridge and lasso. This combination helps address issues related to multicollinearity by constraining the coefficients of correlated variables while also minimizing model overfitting. On the other hand, RF is an ensemble learning method based on decision trees. Unlike ENR, random forest can capture complex nonlinear relationships and interactions between variables without having to specify them in advance. Given that these two methods rely on different algorithms (i.e., penalized regression vs decision tree) for selecting variables, assessing their importance, and generating predictions, comparing them can help to determine which one provides the most accurate predictions in a given context.

To assess model performance and generalizability while minimizing overfitting, we implemented a nested cross-validation (CV) procedure using the *nestedcv* package in R (Lewis et al., 2023). A full description of the nested CV procedure is provided in **section S3** in the Supplement. We evaluated model accuracy using R^2^ (the square of the correlation coefficient) and RMSE. R^2^ was our primary metric because we wanted to prioritize the model’s ability to detect increases in negative emotional states, reflected in corresponding increases in predicted scores. While a stronger correlation (and thus higher R^2^) indicates better alignment between predicted and observed values, it does not necessarily imply lower average prediction error, which is captured by the RMSE.

Initially, we ran nomothetic models using features extracted from all three NLP approaches, including all subjects for group-level analysis. These models identified common predictors of negative emotional states at the group level. To evaluate how well these group-level models generalized to individual participants, we calculated individual metrics, such as R^2^, derived from the nomothetic models for each participant. Subsequently, we employed fully idiographic models, building separate models for each participant to capture person-specific language-emotion associations. These models focused exclusively on within-person variability, aiming for highly individualized predictions. In these idiographic models, variables with low variance were removed based on standard frequency criteria, where the most common value for that variable could not exceed 95% of the total observations. Finally, we compared the three abovementioned approaches, to understand their relative strengths in predicting negative emotions.

#### Feature importance

To evaluate feature importance, we used SHAP (SHapley Additive exPlanations) values, which quantify the contribution of each feature to the model’s predictions in a consistent and interpretable manner by assessing how variations in a feature impact the model’s output^50^. SHAP values were calculated using the R packages *fastshap* and *ggbeeswarm*, which facilitates visualization and interpretation of feature contributions. This method allowed us to identify the relative importance of predictors in the model and their specific effects on the predicted outcomes.

## Figures and Tables

**Table 1.** Demographic and Clinical Characteristics of the Sample

Sample Characteristics *(N=* 97)		
	*n*	%
**Biological Sex**		
Female	75	77.3
Male	22	22.7

**Race**		
American Indian	0	0
Asian	13	13.4
Black	10	10.3
Pacific Islander	1	1.0
White	65	67.0
Multiracial	5	5.2
Other race	3	3.1

**Ethnicity**		
Hispanic	4	4.1
Not Hispanic	92	94.8
Unknown	1	1.0

**Current Diagnoses (DSM-V)**		
MDD	21	21.6
GAD	17	17.5
SAD	5	5.2
Panic Disorder	3	3.1
Specific Phobia	2	2.1
ADHD	1	1.0
PTSD	3	3.1

**Medication**		
Psychotropic Medication	8	8.2

	M (Range)	SD
**Age (years)**	16.2 (12 – 18)	1.9
**Family Income (USD)**	198,895 (0 – 500,000)	111,478

**Note**. MDD = Major Depressive Disorder; GAD = Generalized Anxiety Disorder; SAD = Social Anxiety Disorder; PTSD = Post-Traumatic Stress Disorder; ADHD = Attention-Deficit/Hyperactivity Disorder.

**Table 2.** Comparison of Idiographic, Nomothetic, and Nomothetic–Idiographic Model Performance for Negative Affect, Sadness, Anger, and Nervousness Using Random Forest and Elastic Net

		Nomothetic Group-Level Performance			Nomothetic Per-Participant Performance			Idiographic		
		R^2^	R	RMSE	R^2^	R^2^ range	RMSE	R^2^	R^2^ range	RMSE
**Negative Affect**	Random Forest	.38	.62	0.55	.10 (.10)	.00; .41 Sig.n=53/97	0.52 (0.22)	.10 (.12)	.00; .47 Sig.n=42/97	0.47 (0.20)
	Elastic net	.17	.41	0.64	.11 (.09)	.00; .39 Sig.n=56/97	0.61 (0.27)	.10 (.12)	.00; .53 Sig.n=47/97	0.48 (0.20)
**Sad**	Random Forest	.33	.58	0.75	.10 (.12)	.00; 0.53 Sig.n=48/97	0.73 (0.27)	.10 (.13)	.00; .46 Sig.n=38/94	0.68 (0.22)
	Elastic net	.14	.37	0.85	.09 (.09)	.00; .42 Sig.n=51/97	0.82 (0.32)	.10 (.11)	.00; .51 Sig.n=46/94	0.69 (0.23)
**Angry**	Random Forest	.20	.45	.69	.09 (.12)	.00; .69 Sig.n=37/90	0.63 (0.33)	.06 (.07)	.00; .33 Sig.n=27/85	0.64 (0.29)
	Elastic net	.13	.36	.72	.07 (.07)	.00; .45 Sig.n=49/90	0.88 (0.32)	.07 (.08)	.00; .43 Sig.n=39/85	0.66 (0.31)
**Nervous**	Random Forest	.24	.49	.85	.06 (.08)	.00; .44 Sig.n=33/95	0.81 (0.29)	.08 (.12)	.00; 0.71 Sig.n=33/94	0.75 (0.27)
	Elastic net	.11	.33	.92	.07 (.07)	.00; .35 Sig.n=42/95	0.88 (0.33)	.09 (.12)	.00; 0.84 Sig.n=43/94	0.76 (0.29)

**Table 3.** Comparison of Idiographic Model Performance for Negative Affect, Sadness, Anger, and Nervousness Using LIWC+VADER, LDA, and GPT

		LIWC+VADER			LDA			GPT		
		R^2^	R^2^ range	RMSE	R^2^	R^2^ range	RMSE	R^2^	R^2^ range	RMSE
**Negative Aflect**	Random Forest	.05 (.08)	.00; .42 Sig.n=31/97	0.49 (0.21)	.07 (.09)	.00; .48 Sig.n=36/97	0.49 (0.21)	.10 (.08)	.00; .34 Sig.n=55/97	0.85 (.20)
	Elastic net	.07 (.10)	.00; .55 Sig.n=36/97	0.49 (0.22)	.06 (.07)	.00; .44 Sig.n=35/97	0.50 (0.22)			
**Sad**	Random Forest	.05 (.08)	.00; .38 Sig.n=24/95	0.70 (0.25)	.05 (.08)	.00; .46 Sig.n=25/95	0.71 (0.26)	.10 (.10)	.00; .54 Sig.n=55/96	1.11 (0.26)
	Elastic net	.07 (.08)	.00; .44 Sig.n=42/95	0.70 (0.26)	.07 (.07)	.00; .29 Sig.n=45/95	0.71 (0.27)			
**Angry**	Random Forest	.04 (.06)	.00; .28 Sig.n=15/70	0.72 (0.29)	.03 (.05)	.00; .58 Sig.n=12/85	0.68 (0.32)	.09 (.10)	.00; .44 Sig.n=45/90	0.93 (0.34)
	Elastic net	.05 (.07)	.00; .27 Sig.n=26/70	0.73 (0.31)	.07 (.07)	.00; .28 Sig.n=43/84	0.67 (0.32)			
**Nervous**	Random Forest	.04 (0.06)	.00; .29 Sig.n=19/86	0.80 (0.26)	.05 (.08)	.00; .53 Sig.n=29/94	0.77 (0.28)	.06 (.06)	.00; .29 Sig.n=40/95	1.36 (0.22)
	Elastic net	.05 (.07)	.00; .35 Sig.n=25/86	.81 (28)	.05 (.05)	00; .23 Sig.n=36/94	0.76 (0.28)			

**Table 4.** The 15 most frequent words within the six topics extracted using LDA

Topic number	Topic interpretation	
1	**Social & Evening Activities:** focused on social interactions and nighttime activities with friends	friend, talk, sleep, last, night, last_night, see, time, morning, hang, wake, late, talk_friend, boyfriend, hang_friend,
2	**Academic Life:** centered on school-related activities and academic responsibilities	homework, school, think, class, finish, test, now, take, tomorrow, math, studi, right, finals, essay, stress
3	**Home activities:** capturing leisure activities, particularly around media consumption and family time	watch, nothing, eat, dinner, play, game, show, family, movie, tv, ate, video, eat_dinner, watch_tv, favorite, watch_movie
4	**Family Interactions:** reflecting family relationships and dynamics	mom, think, fun, sister, new, dad, brother, room, read, make, made, book, something, fight, clean
5	**Daily Activities:** representing routine daily activities like meals	go, went, home, walk, outside, lunch, food, drive, music, back, listen, shop, car, hurt, around, dog,
6	**Personal States & Obligations:** describing emotional states, needs, and work-related responsibilities	get, work, feel, want, today, day, need, good, think, just, felt, like, done, tired, sick,
